# Supplementary material for: Group linear non-Gaussian component analysis with applications to neuroimaging
Source: Comput Stat Data Anal. Author manuscript; Available in PMC 2022 Aug 19. (PMC9390952; doi:10.1016/j.csda.2022.107454)
Supplement: Appendix A. Supplementary material [file NIHMS1812434-supplement-Appendix_A__Supplementary_material.pdf]

Web-based Supplementary Materials for "Group linear non-Gaussian component analysis with applications to neuroimaging" by Yuxuan Zhao,

David S. Matteson, Stewart H. Mostofsky, Mary Beth Nebel, and Benjamin B. Risk

S.1 Simulation details

S.1.1 Experimental setup

We used the time courses estimated from group LNGCA of the real data application to estimate a time series model for use in the simulations. For each of the 85 non-Gaussian components extracted for each of the 342 subjects, we first estimated the time courses by ordinary least squares, and then fitted the estimated time courses using an AR(1) process. The median of the estimated coefficient is 0.37 over all component/subject combinations (85*342). We used this AR coefficient to simulate time courses following an AR(1) process in our simulations.

S.1.2 Details on dimension estimation

In this section, we provide the results of dimension estimation. We first show the empirical p-values increase as the dimension increases in Fig. S.1.

Next, we examine the methods under low and high SVAR. The performance of each method is similar to its performance in the medium SVAR setting, again with a tendency for underestimation in some iterations of FOBI-GRF and overestimation in some iterations of FOBIasymp and

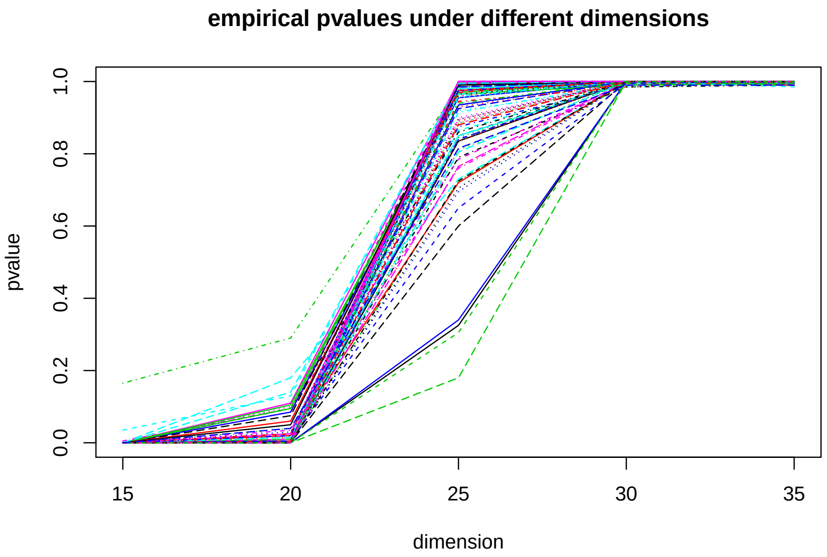

Figure S.1: Each curve connects the p values at 5 points (dimension 15, 20, 25, 30 and 35) for a subject. There are 100 subjects plotted in total. The obtained p values monotonically increase with respect to the dimensions.

FOBIboot, displayed in Fig. S.2. We conjecture the reason is that the variance does not drive the dimension estimation accuracy, but rather the difference in the non-Gaussianity of the non-Gaussian components and the non-Gaussianity achieved by rotating the spatially correlated Gaussian noise.

S.2 Additional simulation results

S.2.1 Robustness to misspecified number of group components

In this section, we provide the results of group LNGCA and group ICA on group components in simulations when the number of group components is misspecified: $q_G = 4$ and $q_G = 2$.

First when $q_G = 4$, both methods perform similar to the case when $q_G = 3$: the three components matched to true group components have similar correlation as when $q_G = 3$, showed in Fig. S.3. The estimated signals from the repetition associated with the median matching error (with true signal) in each setting are depicted in Fig. S.4.

When $q_G = 2$, both methods extract two components with higher variance. The correlation between true group components and their matched estimation are similar to the corresponding

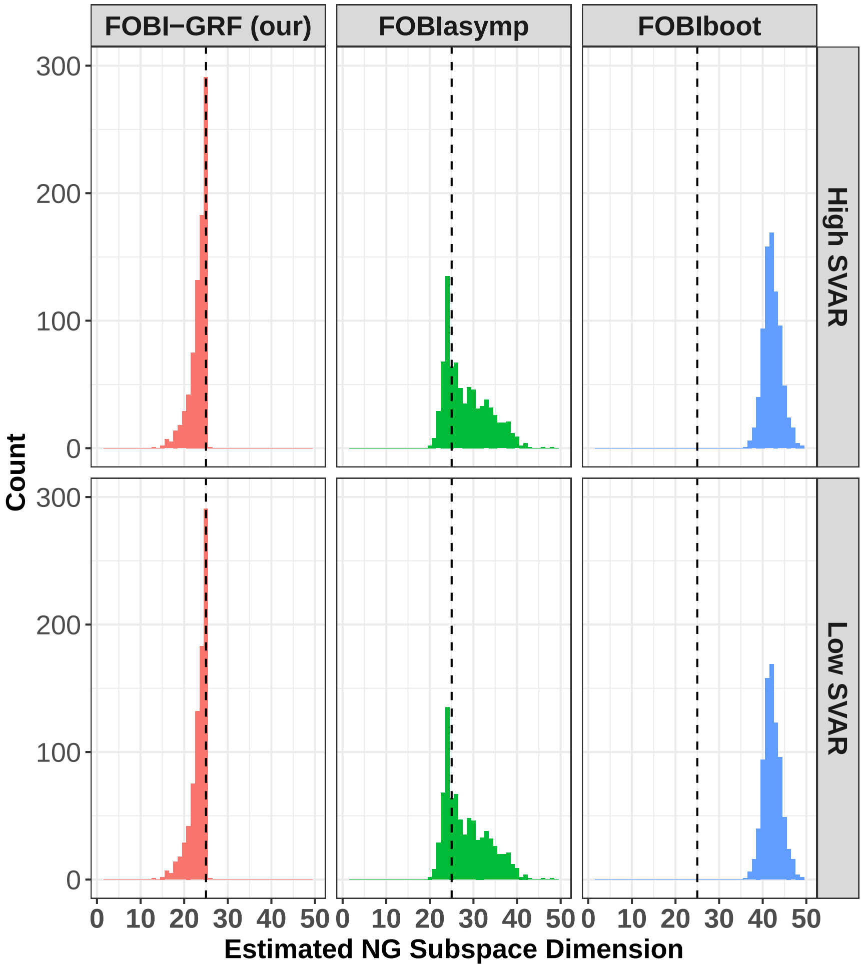

Figure S.2: Estimated non-Gaussian subspace dimension across 800 subjects under high and low SVAR setting. The significance level $\alpha = 0.05$. Dashed line indicates true dimension 25. The most frequently selected dimension using our test, FOBI-GRF, corresponds to the true dimension. Although the test underestimated the dimensions in many simulations, this was due to possibly missing individual components, while it always retained the group components.

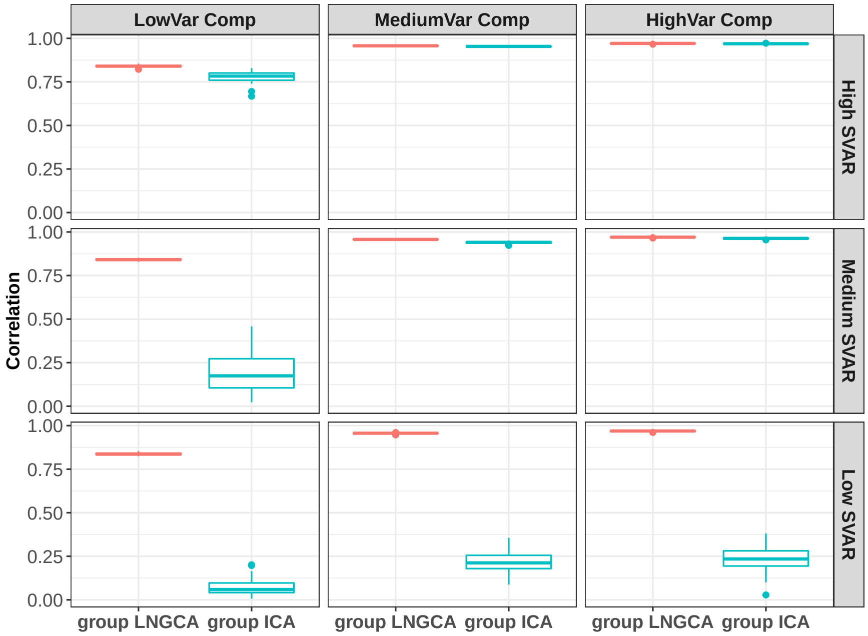

Figure S.3: Correlation between three true group components and their matched estimated components when $q_G = 4$. The correlation under all settings for group LNGCA concentrates at a high correlation value with vanishing variance.

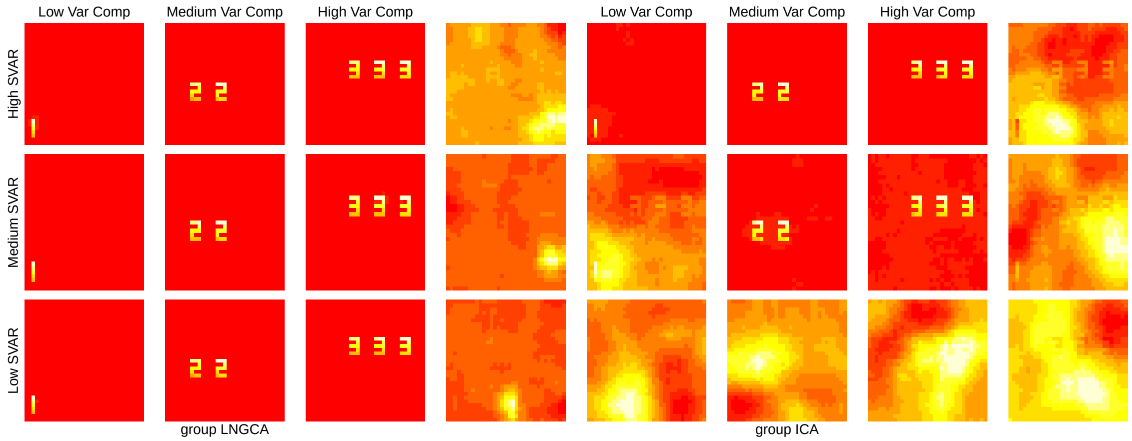

Figure S.4: The estimated group components from a representative simulation (median matching error) when $q_G = 4$. Left four columns display results from group LNGCA, while right four columns display results from group ICA. For each method, the allocated variance increases from left to right among three signals excluding the last estimated noise signal. Three rows represents high, medium and low SVAR settings respectively, from top to bottom.

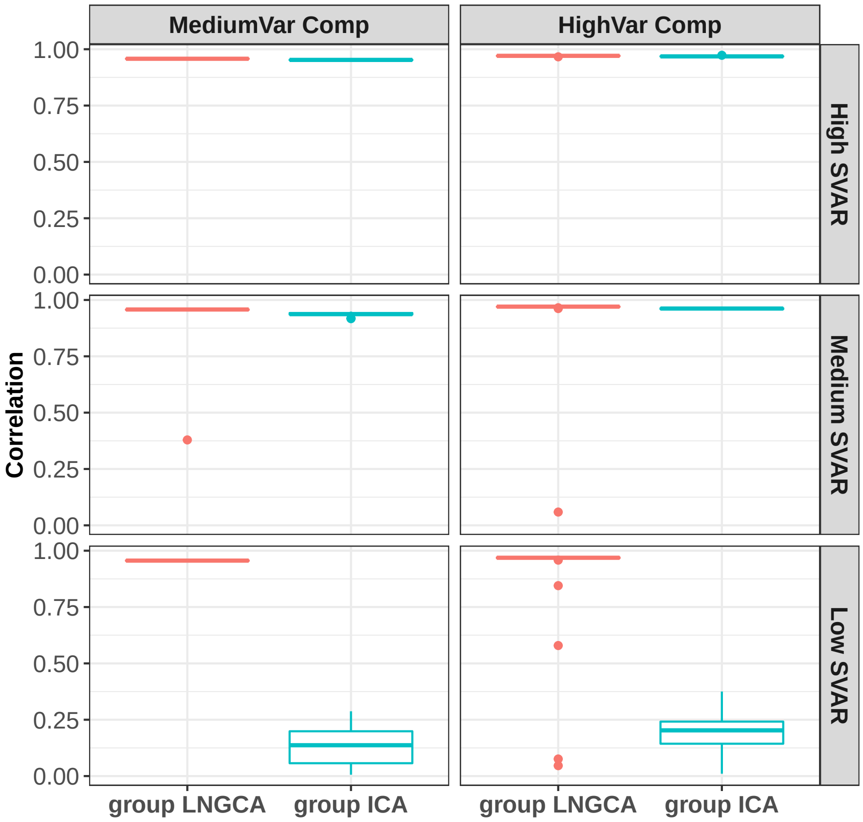

Figure S.5: Correlation between two estimated components and their matched true group components when $q_G = 2$. The correlation under all settings for group LNGCA concentrates at a high correlation value with vanishing variance.

value when $q_G = 3$, showed in Fig. S.5. The estimated signals from the repetition associated with the median matching error (with true signal) in each setting are depicted in Fig. S.6.

S.2.2 Robustness to the number of time points

To examine the robustness of our method to varying number of time points, we also conduct our simulations with $T = 30$ and $T = 70$. We keep all settings fixed except for the number of Gaussian components: 5 Gaussian components for $T = 30$ and 45 Gaussian components for $T = 70$. Note we again use 3 group components and 22 individual NG components.

The results with $T = 30$ are very similar to our original setting ($T = 50$), except that group ICA tends to perform worse at recovering the low variance group component. The results for $T = 70$ are again similar to $T = 50$. Group ICA still performs poorly for the low variance component, since this component is still removed by the PCA step.

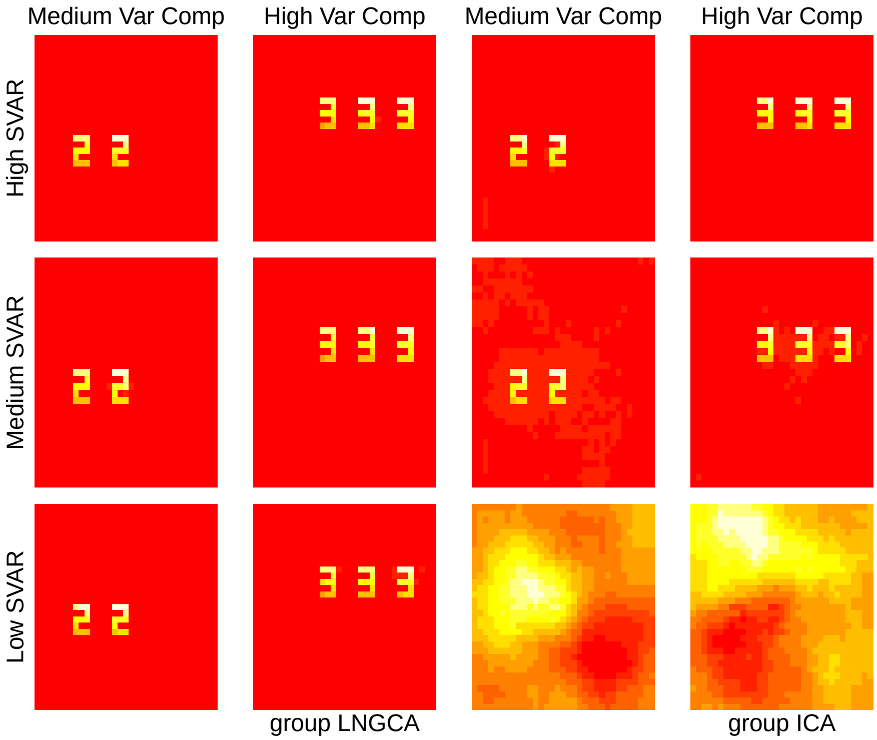

Figure S.6: The estimated group components from a representative simulation (median matching error) when $q_G = 2$. Left two columns display results from group LNGCA, while right two columns display results from group ICA. For each method, the allocated variance increases from left to right. Three rows represents high, medium and low SVAR settings respectively, from top to bottom.

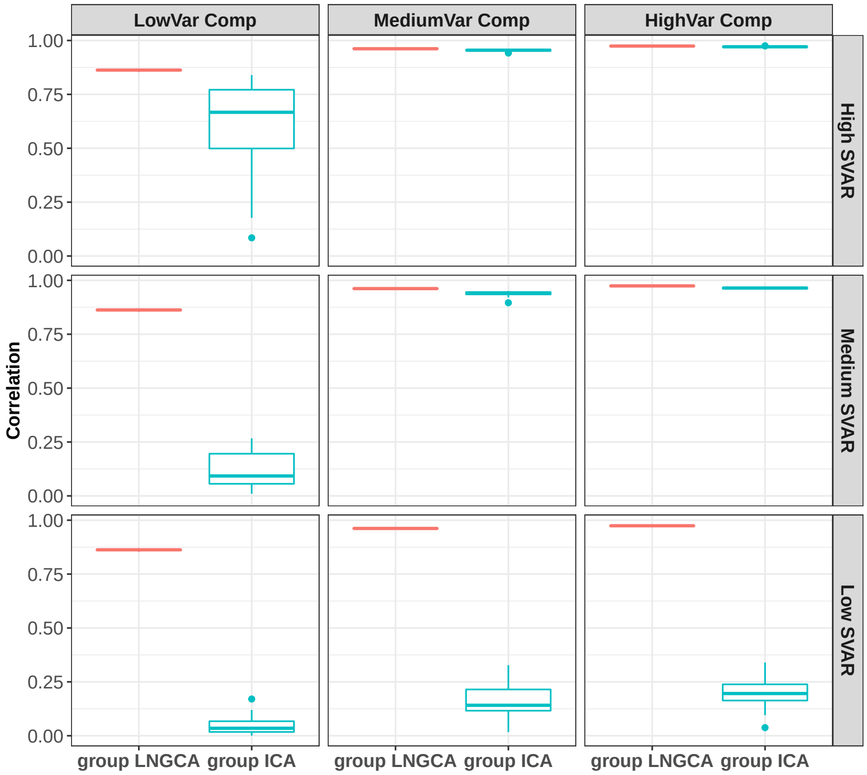

Figure S.7: Correlation between estimated components and their corresponding true components for $T = 30$ with 3 group components, 22 individual components and 5 Gaussian components.

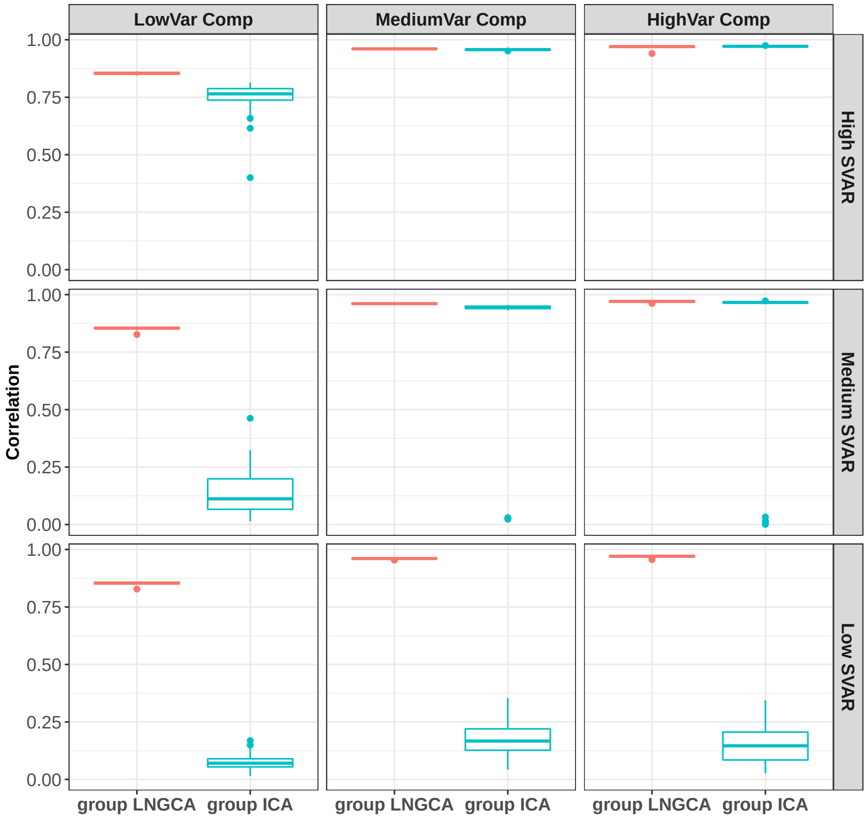

Figure S.8: Correlation between estimated components and their corresponding true components with $T = 70$ when there are 3 group components, 22 individual components and 45 Gaussian components.

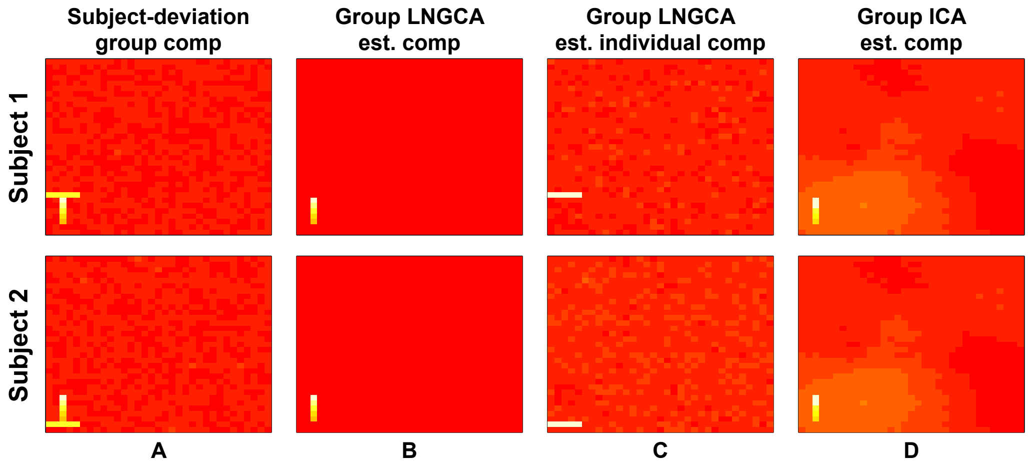

Figure S.9: The first true group component is identical in 18 subjects (Figure 1 of main manuscript, top left panel) but has subject-specific deviations in two subjects, as indicated in column A. Group LNGCA successfully extracts the group components (column B, note rows 1 and 2 are equal). Group LNGCA allocates the subject-specific deviations to individual components (column C). Group ICA also extracts the group component, albeit with less accuracy (column D, again rows 1 and 2 are equal).

S.2.3 Decomposition of subject deviations from group signals

We conduct one repetition of our high SVAR simulation setting according to the previous SVAR setting except we add subject-specific deviations in two subjects: one has extra active pixels on the top of the "1" component, and one has extra active pixels on the bottom of the "1" component, as in Fig. S.9. The estimated group signal from group ICA is slightly worse than that from group LNGCA. We plot the individual components from the two subjects that have the highest correlation with the true subject-deviation component. We see group LNGCA successfully captures the individual deviations with relatively high correlation (0.6).

We describe an approach to identify the individual components that represent subject-specific deviations from the group components. We refer to the components from the initial subject-level LNGCA (Step 1 of Algorithm 1) as the separate-subject components. We refer to the individual components estimated from the group LNGCA procedure (Step 4 of Algorithm 1) as the individual components. For simplicity, consider group component 1 and subject 1. Calculate the correlation between group component 1 with all of subject 1's separate-subject components and select the best match. Call this component 1-A (the estimate of row 1 column A in Fig. S.9). This is an estimate of the group component plus the subject-specific deviation. Next, correlate component

1-A with all of subject 1's individual components and select the individual component with the highest correlation. Then this individual component is an estimate of subject 1's deviation from the group component.

We apply this approach to the above simulation. Fixing group component 1 for subject 1, we find a clear best separate-subject component match, with a correlation of 0.68 to group component 1, whereas the correlations between group component 1 and all other separate-subject components are < 0.04. We call this component 1-A. Next, we correlate the individual components with 1-A and again find a clear best match with a correlation equal to 0.73, whereas the correlations over all other 21 individual components are < 0.02. Thus, the subject 1 deviation from group component 1 is identified and corresponds to row 1 column C in Fig. S.9. Similarly, for component 1 in subject 2, this procedure identifies the individual component depicted in row 2 column C.

S.3 Details on resting-state fMRI data example

S.3.1 Additional information on the data and preprocessing

All children completed a mock scan to acclimate to the scanning environment. Participants were instructed to relax, fixate on a cross-hair, and remain as still as possible. Functional data were preprocessed using SPM12 and custom MATLAB code (`https://github.com/KKI-CNIR/CNIR-fmri_preproc_toolbox`). Rs-fMRI scans were slice-time adjusted using the slice acquired in the middle of the TR as a reference, and rigid body realignment parameters were estimated to adjust for motion. The volume collected in the middle of the scan was non-linearly registered to Montreal Neurological Institute (MNI) space using the MNI EPI template. The estimated rigid body and nonlinear spatial transformations were applied to the functional data in one step, producing 2-mm isotropic voxels in MNI space. Voxel time series were linearly detrended. Data were excluded for between-volume translational movements $>$ 3-mm or rotational movements $>$ 3 degrees.

Group ICA and its PCA steps were applied using GIFT. The second stage PCA was implemented using multi-power iterations (Rachakonda et al., 2016).

Table S.1: Details on dimension test of six subjects

	Sequential Hypothesis Path, presented as dimension(p-value)	Time (mins)
#1	85(0.02)-43(0.03)-**64(0.04)**-75(0.11)-70(0.14)-67(0.10)-66(0.06)-**65(0.07)**	94
#2	85(0.72)-43(0.07)-22(0)-33(0.01)-**38(0.06)**-36(0.04)-**37(0.03)**	134
#3	85(0.01)-106(0.13)-96(0.11)-91(0.11)-**88(0.04)**-90(0.1)-**89(0.12)**	54
#4	85(0.7)-43(0.02)-64(0.27)-54(0.14)-49(0.06)-**46(0.03)**-48(0.08)-**47(0.06)**	200
#5	85(0.11)-43(0)-64(0.03)-75(0.08)-70(0.02)-**73(0.08)-72(0.04)**	151
#6	85(0.06)-43(0)-64(0.03)-75(0.04)-80(0.03)-**83(0.06)-82(0.04)**	149

S.3.2 Dimension estimation

We applied the NG subspace dimension test in Section 2.3 to six participants. The estimated dimension was 65, 38, 89, 47, 73 and 83, corresponding to participants with 128 time points for first three participants and 156 for last three participants. We report the sequential hypothesis applied, its associated p-values and computation time here for each participant in Table S.1. For each participant, the sequential test starts from 85, and the estimated dimension is determined by the two bold test results.

We also implemented a sequential test with `FOBIasymp` and the estimated dimensions are 126, 126, 126, 148, 151 and 153 for participants #1,...,#6 correspondingly. Such large dimension will not help reduce much computation in practice. It also implies `FOBIasymp` tends to overestimate the number of non-Gaussian components, as discussed in our simulation.

S.3.3 Subject-specific components

Example subject-specific components are depicted in Figure S.10.

References

Rachakonda, S., Silva, R. F., Liu, J., and Calhoun, V. D. (2016). Memory efficient pca methods for large group ica. *Frontiers in neuroscience*, 10:17.

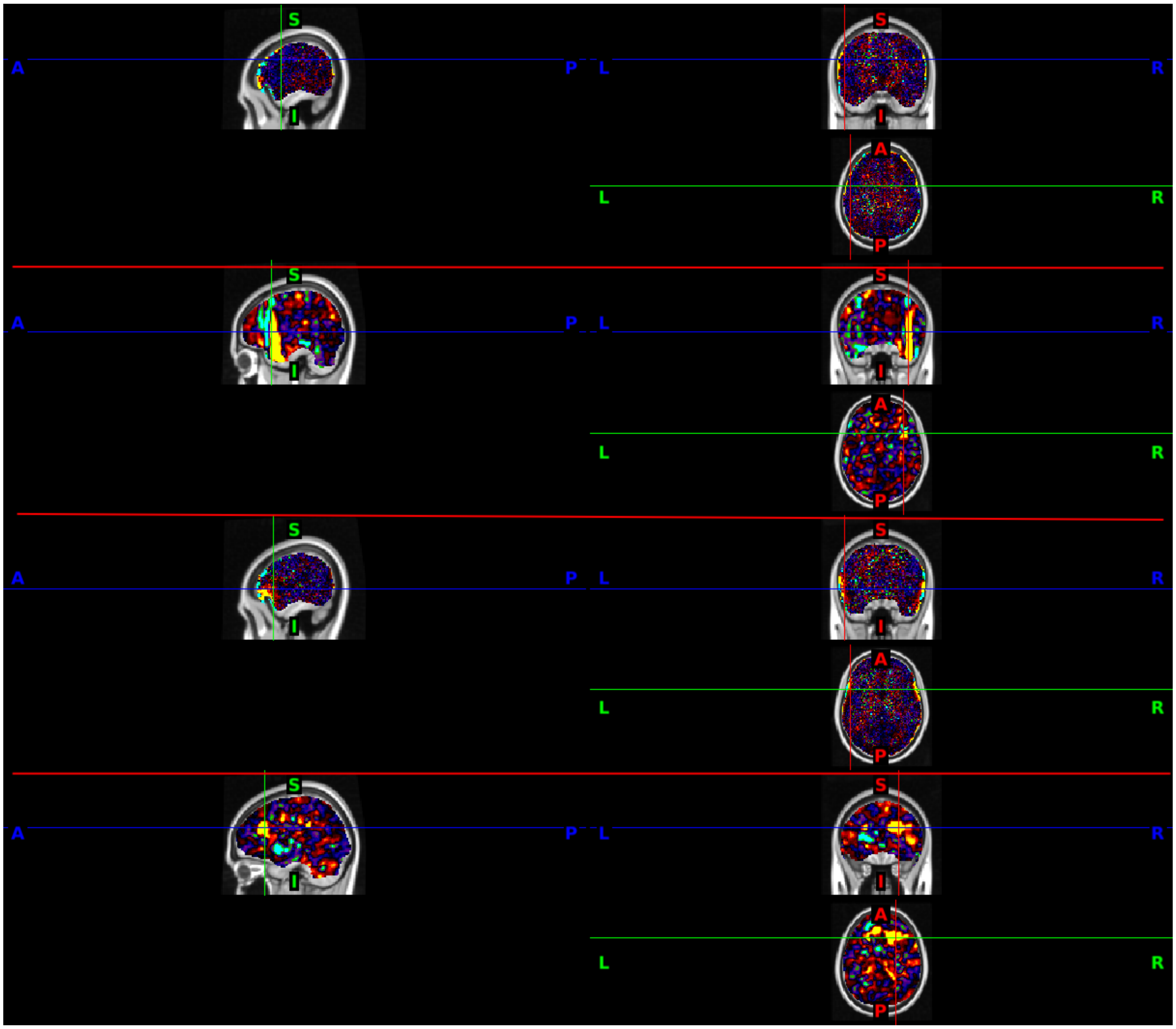

Figure S.10: Example subject-specific (individual) components from four different participants. These components include artifacts. Activation near the brain edge, as in the first and third rows, is often indicative of a motion artifact.
